# Type 2 Diabetes Is a Risk Factor for Suffering and for in-Hospital Mortality with Pulmonary Embolism. A Population-Based Study in Spain (2016–2018)

**DOI:** 10.3390/ijerph17228347

**Published:** 2020-11-11

**Authors:** Rodrigo Jiménez-García, Romana Albaladejo-Vicente, Valentin Hernandez-Barrera, Rosa Villanueva-Orbaiz, David Carabantes-Alarcon, Javier de-Miguel-Diez, José Javier Zamorano-Leon, Ana Lopez-de-Andres

**Affiliations:** 1 Department of Public Health & Maternal and Child Health, Faculty of Medicine, Universidad Complutense de Madrid, 28040 Madrid, Spain; rodrijim@ucm.es (R.J.-G.); mrvillan@med.ucm.es (R.V.-O.); dcaraban@ucm.es (D.C.-A.); josejzam@ucm.es (J.J.Z.-L.); 2 Preventive Medicine and Public Health Teaching and Research Unit, Health Sciences Faculty, Rey Juan Carlos University, Alcorcón, 28922 Madrid, Spain; valentin.hernandez@urjc.es (V.H.-B.); ana.lopez@urjc.es (A.L.-d.-A.); 3 Respiratory Department, Hospital General Universitario Gregorio Marañón, Facultad de Medicina, Universidad Complutense de Madrid, Instituto de Investigación Sanitaria Gregorio Marañón (IiSGM), 28009 Madrid, Spain; javier.miguel@salud.madrid.org

**Keywords:** type 2 diabetes, pulmonary embolism, hospitalization, mortality, incidence

## Abstract

(1) Background: The relationship between type 2 diabetes (T2DM) and pulmonary embolism (PE) has not been well stablished so far. We aim to analyze incidence, clinical conditions and in-hospital mortality (IHM) according to the presence of T2DM among patients hospitalized for suffering from PE. The factors associated with IHM were identified. (2) Methods: Patients aged ≥40 years hospitalized for PE from 2016 to 2018 included in the Spanish National Health System Hospital Discharge Database were analyzed. Dependent variables included incidence, IHM and length of hospital stay. Independent variables were age, sex, diagnosed comorbidities, thrombolytic therapy and inferior vena cava filter placement. Poisson and logistic regression models were constructed for multivariable analysis. (3) Results: Of the 47,190 hospitalizations for PE recorded, 16.52% had T2DM. Adjusted incidence of PE was higher among T2DM women (IRR 1.83; 95% CI: 1.58–1.96) and men (IRR 1.22; 95% CI: 1.18–1.27) than among non-diabetic subjects. Crude IHM in T2DM patients with PE was similar in both sexes but higher than in non-diabetic patients. Among T2DM patients with PE, risk factors for IHM included older age, comorbidity, atrial fibrillation and massive PE. Obesity was associated with lower IHM. Suffering T2DM was a risk of IHM (OR 1.15; 95% CI 1.05–1.26) after PE. (4) Conclusions: The incidence of PE is higher in T2DM men and women than in non-diabetic patients. T2DM was a risk factor for IHM after PE.

## 1. Introduction

The hyperglycemia found in patients with diabetes causes impaired fibrinolysis and elevation of coagulation, factors that increase the likelihood of thrombosis resulting in venous thromboembolism (VTE) [1,2]. Pulmonary embolism (PE) is a potentially life-threatening consequence of VTE [3].

Several authors have found that diabetes sufferers may have higher risk of PE and also have worse outcomes than those without diabetes [4,5,6]. A population-based study in Spain showed that men and women with type 2 diabetes mellitus (T2DM) had significantly higher risk of being hospitalized with PE than the non-diabetic population [7]. Recently, Gupta et al. [8] concluded that diabetes is an independent risk factor for of all-cause mortality in patients who suffer from PE. However, other studies have suggested that the observed association between diabetes and PE could be mainly explained by diabetes-associated comorbid conditions [9,10]. 

Real-world data, using representative population-based information, are necessary and useful to understand the management of PE patients, particularly in diabetic patients [11]. However, existing data from clinical practice provide only limited information on some aspects, such as PE incidence and mortality. 

In this study, we aim to: (i) analyze incidence, clinical conditions and in-hospital mortality (IHM) according to the presence of T2DM among patients hospitalized for suffering from PE; and (ii) identify which study variables are independently associated with IHM.

## 2. Materials and Methods

### 2.1. Design, Setting and Participants

This is an observational retrospective population-based epidemiological study. We used the Hospital Discharge Records of the Spanish National Health System (RAE-CMBD) from 1 January 2016 to 31 December 2018 as the database. The RAE-CMBD uses International Classification of Disease, 10th Revision (ICD-10) to codify up to 20 diagnoses and 20 procedures for each hospital admission. Details on the RAE-CMBD can be found elsewhere [12].

We selected all hospital admissions of patients aged ≥40 years who were hospitalized with a PE diagnosis using the algorithms described by Smith et al. [13]. Briefly, all patient with a primary discharge diagnosis of PE, deep venous thrombosis (DVT) or respiratory failure, in these last two cases with a secondary diagnosis of PE, were considered as PE sufferers [13]. Patients with PE secondary to obstetrical complications, septic PE or iatrogenic PE and those with a code for acute cor pulmonale were excluded [13]. 

Patients with any E11.x (ICD-10 codes) in any diagnosis position of their discharge report were classified as “T2DM patients”, while the rest of the study population were considered “Non-T2DM patients”. Patients with a code, in any diagnosis position, for type 1 diabetes mellitus (ICD-10 codes: E10.x) were excluded.

### 2.2. Study Variables

Dependent variables included incidence, IHM and length of hospital stay (LOHS).

We estimated incidence rates of hospital admissions for PE per 100,000 individuals with and without T2DM. The methods used to calculate incidences according to diabetes status have been described before [7] and are based on data obtained from the Spanish National Health Survey 2017 and the populations estimates of the Spanish National Statistics Institute [14,15]. 

Independent variables included demographic characteristics (age and sex), diagnosed comorbidities and therapeutic procedures. To assess comorbidity, we used the Charlson comorbidity index (CCI) [16]. The CCI includes the following conditions: acute myocardial infarction, congestive heart failure, peripheral vascular disease, cerebrovascular disease, dementia, chronic obstructive pulmonary disease (COPD), rheumatoid disease, peptic ulcer disease, mild and moderate/severe liver disease, hemiplegia/paraplegia, renal disease, cancer and metastatic solid tumor and AIDS/HIV. These conditions were identified using the method described by Quan et al. for ICD-10 administrative databases [16].

We also show the specific prevalence of the following conditions: valvular heart disease, atrial fibrillation (AF), hypertension, obesity and coagulopathy. Massive PE was defined if the patient had codes for any of the following procedures or diagnosis: mechanical ventilation, vasopressors medication or non-septic shock, in any of the procedures (1–20) or diagnosis fields (2–20), as described by Smith et al. [13]. We had to use this algorithm because the ICD-10 does not include a code for severe PE.

We used the diagnosis-related groups codes to identify patients who had undergone a surgical procedure [12].

The therapeutic procedures identified were thrombolytic therapy and inferior vena cava (IVC) filter placement. 

The ICD-10 codes used for these procedures and conditions are shown in Table S1.

### 2.3. Statistical Methods

The RAE-CMBD is a mandatory registry meaning that all Spanish hospitals, public and private, are legally bound to send data of every hospitalization. It is estimated that over 98% of hospital discharges are collected by the RAE-CMBD. As we included all the hospital discharges with a diagnosis of PE, no sample size calculation was required [12].

We described and analyzed the study population stratified by sex. 

Categorical variables are shown as proportions, and continuous variables are shown as medians with interquartile ranges (IQR) or as means with standard deviations (SD), as appropriate. The *t*-test, Mann–Whitney test, Kruskall–Wallis test and bivariate unconditional logistic regression were used to compare patients with and without T2DM.

The very large samples used in our investigation may result on finding statistically significant results even when the magnitude of these differences is small. To assess the relevance of associations besides the sample size, we calculated the effect sizes using Cramer’s and Cohen’s tests. According to Cohen’s recommendations, we consider relevant differences if two groups differ by an effect size of over 0.2 [17].

To assess differences in the incidence rates, Poisson regression models adjusted by age and/or sex were constructed. Incidence rate ratios (IRR) with 95% confidence intervals (CI) are reported as a measure of association.

Multivariable logistic regression models were constructed to identify factors associated with IHM. The multivariable logistic regressions were constructed including variables statistically significant in the bivariate analysis and those that, even if not statistically significant, were considered relevant from an epidemiological or clinical viewpoint. We included all variables in the initial model and one at each step, and we decided to eliminate, or not, variables according to their significance in the model used (Wald statistic) and comparing the model’s goodness of fit (Hosmer–Lemeshow statistic) with the previous step using the likelihood ratio test. Once we obtained a final model, we examined the effects of interactions. Results are shown as odds ratios (ORs) with their 95% CI.

All analyses were performed with Stata version 14 (Stata, College Station, TX, USA).

### 2.4. Ethical Aspects

The RAE-CMBD is a retrospective de-identified database that was provided to us free of charge by the Spanish Ministry of Health. The Spanish legislation determines that this type of investigation with public access databases does not need approval by an ethics committee. 

## 3. Results

We analyzed 47,190 hospitalized patients aged ≥40 years with a PE diagnosis in Spain (2016–2018). T2DM was coded in 16.52% of the total sample (3541 men and 4258 women). Primary diagnosis of PE in patients with T2DM was 92.15% and in non-T2DM patients 94.91%, and similar figures were found in men and women (all *p* < 0.001) (Table 1).

### 3.1. Incidence of Pulmonary Embolism According to T2DM Status

The observed incidence of PE was higher in people with T2DM than in non-diabetic people (84.96 cases per 100,000 T2DM population vs. 57.99 cases per 100,000 non-T2DM population; *p* < 0.001). Crude incidences of PE in women and men with T2DM were 98.09 and 73.17 cases per 100,000 T2DM population, respectively, vs. 58.23 and 57.72 cases per 100,000 non-T2DM women and men, respectively (all *p*-values < 0.001) (Table 1). 

Furthermore, after Poisson regression analysis, the adjusted incidence of PE was higher among patients with T2DM for both sexes (women, IRR 1.83; 95% CI 1.58–1.96; men, IRR 1.22; 95% CI 1.18–1.27) and for the entire population including both sexes (IRR 1.40; 95% CI 1.37–1.44).

### 3.2. Clinical Characteristics of Patients Hospitalized with Pulmonary Embolism According to T2DM Status

Women represented 54.59% and 53.13% of T2DM and non-diabetic patients suffering from PE, respectively.

Overall, the mean age was significantly higher among T2DM patients (75.45; SD = 10.84 years) than non-T2DM patients (72.23; SD = 13.58 years), and T2DM patients also had a higher mean CCI (*p* < 0.001). Specifically, men with T2DM have higher prevalence of congestive heart failure (13.89% vs. 8.92%; *p* < 0.001), COPD (25.28% vs. 21.98%; *p* < 0.001) and renal disease (17.9% vs. 8.75%; *p* < 0.001). Women with T2DM have higher prevalence of congestive heart failure (16.65% vs. 12.09%; *p* < 0.001) and renal disease (16.49% vs. 8.82%; *p* < 0.001) (Table 1). When we compare T2DM men with T2DM women, we observe that men are younger, with a higher mean CCI, and have more frequently acute myocardial infarction. chronic obstructive pulmonary disease and cancer, whereas women have more congestive heart failure and dementia. 

Specific comorbid conditions, procedures and in-hospital outcomes of patients hospitalized with PE according to diabetes status are shown in Table 2.

Patients with T2DM have significantly higher prevalence of massive PE (4.5% vs. 3.36%), AF (11.18% vs. 9.18%), hypertension (58.03% vs. 39.32%) and obesity (19.75% vs. 11.09%) (all *p*-values < 0.001). 

No significant differences were found in surgery, thrombolytic therapy or IVC filter placement between patients with and without T2DM in either men or women (Table 2).

The median LOHS was significantly higher in men and women with T2DM than in non-T2DM patients. Crude IHM was 9.12% for T2DM patients and 7.18% for non-T2DM patients (*p* < 0.001).

### 3.3. Distribution of IHM According to Study Covariates for Men and Women Hospitalized with Pulmonary Embolism According to T2DM Status

Table 3 shows IHM according to clinical characteristics among patients with and without T2DM admitted for PE stratified by sex.

As can been seen in Table 3, for admissions with PE as the primary diagnosis, patients with T2DM had higher IHM than patients without T2DM (8% vs. 6.35%; *p* < 0.001). 

Remarkable are the very high IHM results found in T2DM women with hemiplegia/paraplegia (36.84%), congestive heart failure (16.5%) and cancer (15.38%). Among men with T2DM, high values are found among those suffering from dementia (18.44%), cancer (15.74%) and cerebrovascular disease (15.43%) (Table 3).

Patients with T2DM who had valvular heart disease and hypertension have higher values of IHM than patients without T2DM suffering these conditions, as can been seen in Table S2. IHM was significantly higher in women with T2DM who suffered from AF (16.63% vs. 12.19%; *p* = 0.010) and obesity (6.77% vs. 4.17%; *p* = 0.001) and underwent surgery (28% vs. 15.9%; *p* = 0.038) than women without T2DM. 

The crude IHM for men (9.12%) and women (9.11%) with T2DM who suffered from PE was not significantly different. However, it was higher than among non-diabetic men and women (9.12% vs. 7.12%; *p* < 0.001 for men, and 9.11% vs. 7.24%; *p* < 0.001 for women).

### 3.4. Multivariable Logistic Regression Analysis of the Factors Associated with IHM Among Patients Hospitalized with Pulmonary Embolism

Table 4 shows the result of the multivariable analysis of the factors associated with IHM among T2DM patients with PE. Comorbidity was a factor associated with IHM in men and women with T2DM. However, older age (>75 years) was a factor only associated with IHM in men with T2DM (OR 2.48; 95% CI 1.5–4.1).

The presence of massive PE and AF increased the probability of dying in women and men with T2DM. However, obesity was associated with lower IHM in both men and women with T2DM (OR 0.55; 95% CI 0.36–0.85, and OR 0.68; 95% CI 0.51–0.92, respectively).

Finally, after adjusting for possible confounders, the probability of dying for patients hospitalized with PE who suffered T2DM was 15% higher (OR 1.15; 95% CI 1.05–1.26) than for non-diabetic patients. According to sex, among T2DM men, the risk of dying was 1.18 (OR 1.12 95% CI 1.06–1.35) higher than for non-diabetic men, and for T2DM women, the OR was 1.12 (95% CI 1.01–1.29).

## 4. Discussion

This population-based study showed that the incidence of PE was significantly higher in patients with T2DM than in patients with non-T2DM. Furthermore, multivariable Poisson regression confirmed the independent effect of T2DM for both sexes, although in T2DM women, the IRR is almost 2 (1.83) and in T2DM men it is 1.22. The incidence of PE observed in our study is consistent with our earlier findings and with the findings of other authors [7,18]. 

Factors such as the age of the population can affect the observed incidence of PE in different countries [13,19,20,21,22,23,24]. The average age of our population is high (74.34 years). Studies conducted in the US report lower mean ages ranging from 64 to 68 years [13,19]. On the other hand, investigations in European countries with a demographic structure similar to Spain, such as Italy, Germany, France or Denmark, found figures from 71 to 73 years, more in line with our results [20,21,22,23,24]. However, our higher mean age can also be partially explained because we included only a population aged 40 years and older.

In the pathogenesis of VTE, diabetes plays an important role [1,25]. Different studies found that metabolic syndrome is more prevalent in patients with T2DM and the prevalence of VET is higher in patients with metabolic syndrome than in the general population [26,27].

As found in other studies, the incidence of PE is much higher in women than in men both among diabetics and non-diabetics. Raptis et al. [28] also found a female predominance in PE incidence, which was mainly attributed to the age groups above 70 years. One explanation could be the existence of differences in life expectancy between the sexes. Furthermore, differences in thrombotic and fibrinolytic activity between men and women may influence the sex-related discrepancies detected in PE incidence in older age [29]. However, another study found no differences in the annual rates of hospitalization for PE between sexes [30]. 

Our results document that women and men with T2DM had more comorbidities than those without diabetes, consistent with findings of previous studies [7]. Among the comorbidities that have higher prevalence in diabetic patients were atrial fibrillation, hypertension and obesity, some of which are known risk factors for PE [7,31]. 

We found that from 2016 to 2018, under 1% of patients with PE in Spain had undergone an IVC filter placement. Pomero et al. analyzed 60,813 patients hospitalized for a first episode of acute PE over an 11 years’ period in Northwest Italy, finding that only 745 (1.22% of the total population) patients had received an IVC filter placement during the hospital stay. Furthermore, from 2002 to 2012, the use of IVC decreased significantly, reaching the lowest value in 2011 (0.77%) [24]. Our results and those found in Italy disagree with reports from the US using the National Inpatient Sample (NIS) that reported much higher rates of IVC filter placement with figures over 10% for 2014 and with a constant decrease overtime since 2005 [19,32]. 

Bikdeli B et al. suggest that the less frequent utilization of IVC filters in PE patients in the US overtime is likely multifactorial, including doubts with the safety and efficacy of these devices [32]. The reasons for the large differences found between the US and Europe should be investigated.

Regarding the use of systemic thrombolysis, we observed that this procedure was used in 5.5% of patients hospitalized with PE, slightly higher than what was found in the US, where according to the NIS from 2011 to 2014, among the 1,283,063 hospitalizations with PE, systemic thrombolysis use increased from 2.1% to 3.3% (*p* < 0.001 for trend) [33]. A study conducted in another European country reported figures similar to ours [21].

As can be seen in Table S3, the use of both diagnostic procedures was much higher among patients who suffered a massive PE, with figures for thrombolytic therapy of 19.5% for non-diabetic patients and 14.81% for those suffering from T2DM (*p* = 0.045). Coding of IVC filter placement was found in 3.1% of non-diabetic and 1.71% among those suffering from this condition (*p* = 0.161). The greater use of these therapeutic procedures among more severely ill patients has been described before [19,34].

The available findings on the possible association between PE, sex and IHM do not provide a definite conclusion. The results from the United States NIS, 2003–2011, found that women admitted with acute PE, compared with men, tended to have higher IHM (OR: 1.09; 95% CI: 1.03–1.15) [35]. On the other hand, data from Alberta, Canada (2002–2012), reported that in most age groups, women suffering from VTE were less likely to be hospitalized than men [36]. A small-sized, single-center study, focused on a selected group of hospitalized patients with a diagnosis of Alzheimer’s dementia, found that in-hospital mortality due to PE exhibited evident differences by sex, with a significantly higher risk for males (OR:1.76; 95% CI: 1.49–2.08) [37]. In our study, regarding IHM, no significant differences are found by sex in patients with T2DM.

Our results highlight key aspects in IHM. As we expected and previously described, older age, massive PE and higher coexisting comorbidities were variables associated with IHM for women and men with T2DM [8,38,39,40]. 

Like other authors, we found that AF was a risk factor for IHM in patients with PE [39,40,41,42,43]. However other studies have described no significant association between AF and mortality outcomes among PE patients [44,45,46].

Ng et al. [39] analyzed the effect of AF in the IHM for PE according to the presence of this arrhythmia before or after admission with PE. These authors concluded that the worst prognosis was found in patients admitted due to PE with AF diagnosed prior to a PE episode compared with those patients with AF diagnosed after hospitalization [39]. 

As can be seen in Table S4, in our study, most patients had AF present when admitted to the hospital (>87%), with no significant differences in the distribution besides the presence of T2DM. The overall IHM after PE showed similar figures for those with AF present or not at admission (13.53% vs. 14.97%; *p* = 0.346). However, the IHM among T2DM with AF present on admission was significantly higher than among those without diabetes.

The association between AF and PE has pathophysiological bases [39,40,41,42,43]. AF promotes a pro-coagulant state and the blood stasis that occurs in both atria during AF facilitates thrombus formation. NG et al. reported that among patients suffering from PE, those with AF had proportionally significantly higher cardiovascular causes of death compared to those without AF, suggesting that the negative effect of AF is on cardiovascular outcomes in patients with acute PE [39].

Interestingly, obesity reduced the IHM for PE in our investigation. Previous studies conducted in Spain and other counties found that obesity has a protective effect on in-hospital and after-discharge mortality in patients who suffered from PE [31,34,47,48]. El-Menyar et al. suggest that an “obesity survival paradox” that has been previously demonstrated for subjects with other cardiovascular conditions may also affect PE [48]. The activity of the endocannabinoid system has been proposed as a possible mechanism for the reduced mortality in obese patients with pulmonary embolism [47]. However, other studies reached the opposite conclusion with trend towards worse prognostics in patients who are obese [49]. Furthermore, this is reinforced by large autopsy studies, where in each category of above-normal BMIs, individuals who were obese were more likely to die from PE [50,51]. Obesity results in a pro-thrombotic status that alters fibrinolytic activity and the coagulation profile [52]. 

Hainer et al. suggested several reasons to explain the obesity paradox for several cardiovascular conditions including thromboembolism. These include biological factors such as the tumor necrosis factor (TNF)-α, ghrelin and thromboxane A2 and a greater mobilization of endothelial progenitor cells [53]. However, Standl et al. pointed out several arguments to refute the obesity paradox including treatment bias, survival or selection bias, comorbidities and confounders, anabolic deficiency or malnutrition–inflammation syndrome and the prospective evaluation of weight loss/weight changes [54].

The strengths of this investigation are that we analyzed data from an entire country, over a three-year period, collected using a reliable database. Nevertheless, our study is subject to several limitations, some of which are inherent to the study design and the use of administrative data such as the RAE-CMBD. First, it was based on ICD discharge codes rather than clinical criteria, which might be subject to underreporting or miscoding. Nevertheless, the validity of the diagnosis codes for PE are high compared with medical chart review criteria [55]. Second, our data source is limited by the lack of information about the glycosylated hemoglobin, duration of T2DM, chronic complications, severity, burden and treatment of T2DM. What is also relevant is that the use of anticoagulant medication, especially given the large number of patients with AF, is not collected in the register. Third, the cause of death could not be obtained, so we only evaluated all-cause hospital mortality. However, previous studies have demonstrated that IHM is mostly related to acute PE episodes, in contrast to other causes of death, such as comorbidities, which affect mortality over the long term [20]. Fourth, another possible limitation of our investigation is that PE may be secondary to other conditions such as mechanical ventilation, ICU admission or shock. As can be seen in Table 1, over 91% of our study population had PE coded as the principal diagnosis: according to the coding methodology of the RAE-CMBD, the primary diagnosis must be present on admission [12]. Therefore, it is very improbable that a patient will be admitted with another diagnosis and develop a PE within the hospital and have PE coded as the primary diagnosis. The only exceptions to this rule are patients with a principal diagnosis of respiratory failure or deep venous thrombosis and a secondary diagnosis of PE. We reviewed the database and among these 2616 patients with PE in a secondary position, only 71 had a not present on admission (POA) code. This represents 0.15% of our study population, and in our opinion, the effect on the study result would be minimal. 

## 5. Conclusions

In conclusion, using a database from an entire country, we demonstrate that the incidence of PE in persons with T2DM is higher than in non-diabetic subjects for both sexes and older patients are the most affected. The incidence of PE is higher in diabetic women than in diabetic men, however no significant differences in the IHM were found regarding sex. Risk factors of IHM in diabetic patients were older age and comorbidities such as AF and massive PE, however obesity was a predictor of survival. Finally, the presence of T2DM was an independent risk factor for in-hospital death in men and women hospitalized for PE.

## Figures and Tables

**Table 1:** Incidence rates and clinical characteristics among patients hospitalized with pulmonary embolism according to sex and type 2 diabetes (T2DM) status in Spain (2016–2018).

	Men				Women				Both			
	No T2DM	T2DM	*p*	ES	No T2DM	T2DM	*p*	ES	No T2DM	T2DM	*p*	ES
Total, *n* (Inc Rate*100,000 inh)	18,461 (57.72)	3541 (73.17)	<0.001	0.55	20,930 (58.23)	4258 (98.09)	<0.001	0.78	39,391 (57.99)	7799 (84.96)	<0.001	0.63
Age, mean (SD)	68.75 (13.48)	72.68 (11.08)	<0.001	0.34	75.31 (12.91)	77.76 (10.07)	<0.001	0.22	72.23 (13.58)	75.45 (10.84)	<0.001	0.27
Admissions with PE as primary diagnosis, *n* (%)	17,525 (94.93)	3254 (91.89)	<0.001	0.27	19,862 (94.9)	3933 (92.37)	<0.001	0.26	37,387 (94.91)	7187 (92.15)	<0.001	0.26
CCI, mean (SD)	0.86 (0.82)	1.14 (1.03)	<0.001	0.76	0.75 (0.72)	0.91 (0.91)	<0.001	0.71	0.8 (0.77)	1.01 (0.97)	<0.001	0.70
CCI = 0, *n* (%)	7744 (41.95)	1063 (30.02)	<0.001	0.24	9364 (44.74)	1629 (38.26)	<0.001	0.20	17,108 (43.43)	2692 (34.52)	<0.001	0.22
CCI 1–2, *n* (%)	6864 (37.18)	1400 (39.54)	<0.001		8168 (39.03)	1674 (39.31)	<0.001		15,032 (38.16)	3074 (39.42)	<0.001	
CCI >2, *n* (%)	3853 (20.87)	1078 (30.44)	<0.001		3398 (16.24)	955 (22.43)	<0.001		7251 (18.41)	2033 (26.07)	<0.001	
AMI, *n* (%)	624 (3.38)	230 (6.5)	<0.001	0.19	289 (1.38)	105 (2.47)	<0.001	0.15	913 (2.32)	335 (4.3)	<0.001	0.17
CHF, *n* (%)	1646 (8.92)	492 (13.89)	<0.001	0.41	2530 (12.09)	709 (16.65)	<0.001	0.22	4176 (10.6)	1201 (15.4)	<0.001	0.30
PVD, *n* (%)	928 (5.03)	254 (7.17)	<0.001	0.15	455 (2.17)	100 (2.35)	0.479	0.11	1383 (3.51)	354 (4.54)	<0.001	0.13
CVD, *n* (%)	640 (3.47)	188 (5.31)	<0.001	0.16	811 (3.87)	225 (5.28)	<0.001	0.14	1451 (3.68)	413 (5.3)	<0.001	0.14
Dementia, *n* (%)	604 (3.27)	141 (3.98)	0.032	0.02	1732 (8.28)	407 (9.56)	0.006	0.03	2336 (5.93)	548 (7.03)	<0.001	0.02
COPD, *n* (%)	4057 (21.98)	895 (25.28)	<0.001	0.25	2906 (13.88)	605 (14.21)	0.578	0.09	6963 (17.68)	1500 (19.23)	0.001	013
Rheumatoid disease, *n* (%)	292 (1.58)	67 (1.89)	0.182	0.01	771 (3.68)	117 (2.75)	0.003	0.03	1063 (2.7)	184 (2.36)	0.088	0.01
PUD, *n* (%)	81 (0.44)	25 (0.71)	0.035	<0.01	76 (0.36)	21 (0.49)	0.212	<0.01	157 (0.4)	46 (0.59)	0.018	<0.01
HP/PAPL, *n* (%)	105 (0.57)	15 (0.42)	0.283	<0.01	98 (0.47)	19 (0.45)	0.847	<0.01	203 (0.52)	34 (0.44)	0.365	0.01
Cancer, *n* (%)	3993 (21.63)	807 (22.79)	0.126	0.12	3380 (16.15)	611 (14.35)	0.003	0.13	7373 (18.72)	1418 (18.18)	0.267	0.12
Liver disease, *n* (%)	1168 (6.33)	274 (7.74)	0.002	0.15	860 (4.11)	244 (5.73)	<0.001	0.14	2028 (5.15)	518 (6.64)	<0.001	0.14
Renal disease, *n* (%)	1616 (8.75)	634 (17.9)	<0.001	0.37	1845 (8.82)	702 (16.49)	<0.001	0.34	3461 (8.79)	1336 (17.13)	<0.001	0.35
AIDS, *n* (%)	64 (0.35)	8 (0.23)	0.249	<0.01	17 (0.08)	2 (0.05)	0.458	<0.01	81 (0.21)	10 (0.13)	0.155	<0.01

Inc Rate*100,000 inh: incidence rate per 100,000 inhabitants. CCI: Charlson comorbidity index; AMI: acute myocardial infarction. CHF: congestive heart failure. PVD: peripheral vascular disease. CVD: cerebrovascular disease. COPD: chronic obstructive pulmonary disease. PUD: peptic ulcer disease. Liver disease: mild and moderate/severe liver disease. HP/PAPL: hemiplegia/paraplegia. The *p*-value for the difference between patients with T2DM and without T2DM was calculated with the bivariate unconditional logistic regression model. ES: effect size. Effect size > 0.2 is the recommended minimum effect size.

**Table 2:** Prevalence of specific comorbid conditions, procedures and in-hospital outcomes among patients hospitalized with pulmonary embolism (PE) according to sex and type 2 diabetes (T2DM) status in Spain (2016–2018).

	Men				Women				Both			
	No T2DM	T2DM	*p*	ES	No T2DM	T2DM	*p*	ES	No T2DM	T2DM	*p*	ES
Massive PE, *n* (%)	650 (3.52)	188 (5.31)	<0.001	0.24	673 (3.22)	163 (3.83)	0.042	0.16	1323 (3.36)	351 (4.5)	<0.001	0.21
Valvular heart disease, *n* (%)	970 (5.25)	190 (5.37)	0.786	<0.01	1540 (7.36)	293 (6.88)	0.275	<0.01	2510 (6.37)	483 (6.19)	0.554	<0.01
Atrial fibrillation, *n* (%)	1600 (8.67)	391 (11.04)	<0.001	0.22	2018 (9.64)	481 (11.3)	0.001	0.26	3618 (9.18)	872 (11.18)	<0.001	0.23
Hypertension, *n* (%)	6502 (35.22)	1953 (55.15)	<0.001	0.65	8988 (42.94)	2573 (60.43)	<0.001	0.48	15,490 (39.32)	4526 (58.03)	<0.001	0.57
Obesity, *n* (%)	1583 (8.57)	565 (15.96)	<0.001	0.31	2784 (13.3)	975 (22.9)	<0.001	0.40	4367 (11.09)	1540 (19.75)	<0.001	0.36
Coagulopathy, *n* (%)	349 (1.89)	42 (1.19)	0.004	0.14	297 (1.42)	52 (1.22)	0.314	0.01	646 (1.64)	94 (1.21)	0.005	0.08
Undergone surgery, *n* (%)	376 (2.04)	76 (2.15)	0.674	<0.01	346 (1.65)	50 (1.17)	0.022	<0.01	722 (1.83)	126 (1.62)	0.187	<0.01
Thrombolytic therapy, *n* (%)	1078 (5.84)	187 (5.28)	0.191	<0.01	1119 (5.35)	226 (5.31)	0.918	<0.01	2197 (5.58)	413 (5.3)	0.320	<0.01
IVC filter placement, *n* (%)	185 (1)	29 (0.82)	0.309	<0.01	182 (0.87)	27 (0.63)	0.123	<0.01	367 (0.93)	56 (0.72)	0.067	<0.01
LOHS, median (IQR)	7 (5)	7 (6)	<0.001	0.26	7 (6)	8 (6)	<0.001	0.31	7 (6)	8 (7)	<0.001	0.29
IHM, *n* (%)	1314 (7.12)	323 (9.12)	<0.001	0.20	1515 (7.24)	388 (9.11)	<0.001	0.21	2829 (7.18)	711 (9.12)	<0.001	0.20

IVC: inferior vena cava. LOHS: length of hospital stay. IHM: in-hospital mortality. The *p*-value for the difference between patients with T2DM and without T2DM was calculated with the bivariate unconditional logistic regression model. ES: effect size. Effect size > 0.2 is the recommended minimum effect size.

**Table 3:** In-hospital mortality according to clinical characteristics among men and women with and without type 2 diabetes (T2DM) hospitalized with pulmonary embolism in Spain (2016–2018).

	Men			Women			Both		
	No T2DM	T2DM	*p*-Value	No T2DM	T2DM	*p*-Value	No T2DM	T2DM	*p*-Value
40–59 years, *n* (%)	185 (3.68)	21 (4.5)	0.372	137 (4.92)	18 (7.44)	0.091	322 (4.12)	39 (5.5)	0.082
60–74 years, *n* (%)	446 (6.88)	111 (7.91)	0.173	296 (5.23)	69 (6.06)	0.258	742 (6.11)	180 (7.08)	0.067
>75 years, *n* (%)	683 (9.84)	191 (11.44)	0.052	1082 (8.66)	301 (10.46)	0.002	1765 (9.08)	492 (10.82)	<0.001
Admissions with PE as primary diagnosis, *n* (%)	1079 (6.16)	257 (7.9)	<0.001	1294 (6.51)	318 (8.09)	<0.001	2373 (6.35)	575 (8)	<0.001
CCI = 0, *n* (%)	236 (3.05)	45 (4.23)	0.040	350 (3.74)	72 (4.42)	0.186	586 (3.43)	117 (4.35)	0.017
CCI 1–2, *n* (%)	577 (8.41)	135 (9.64)	0.133	707 (8.66)	183 (10.93)	0.003	1284 (8.54)	318 (10.34)	0.001
CCI > 2, *n* (%)	501 (13)	143 (13.27)	0.821	458 (13.48)	133 (13.93)	0.721	959 (13.23)	276 (13.58)	0.681
AMI, *n* (%)	61 (9.78)	26 (11.3)	0.513	32 (11.07)	18 (17.14)	0.112	93 (10.19)	44 (13.13)	0.141
CHF, *n* (%)	262 (15.92)	71 (14.43)	0.425	357 (14.11)	117 (16.5)	0.092	619 (14.82)	188 (15.65)	0.477
PVD, *n* (%)	85 (9.16)	27 (10.63)	0.479	38 (8.35)	10 (10)	0.596	123 (8.89)	37 (10.45)	0.366
CVD, *n* (%)	104 (16.25)	29 (15.43)	0.787	131 (16.15)	34 (15.11)	0.706	235 (16.2)	63 (15.25)	0.645
Dementia, *n* (%)	94 (15.56)	26 (18.44)	0.403	237 (13.68)	59 (14.5)	0.669	331 (14.17)	85 (15.51)	0.421
COPD, *n* (%)	349 (8.6)	98 (10.95)	0.027	189 (6.5)	45 (7.44)	0.402	538 (7.73)	143 (9.53)	0.020
Rheumatoid disease, *n* (%)	11 (3.77)	3 (4.48)	0.787	48 (6.23)	14 (11.97)	0.026	59 (5.55)	17 (9.24)	0.056
PUD, *n* (%)	5 (6.17)	1 (4)	0.683	6 (7.89)	1 (4.76)	0.627	11 (7.01)	2 (4.35)	0.521
HP/PAPL, *n* (%)	19 (18.1)	2 (13.33)	0.651	18 (18.37)	7 (36.84)	0.079	37 (18.23)	9 (26.47)	0.264
Cancer, *n* (%)	554 (13.87)	127 (15.74)	0.167	454 (13.43)	94 (15.38)	0.197	1008 (13.67)	221 (15.59)	0.057
Liver disease, *n* (%)	90 (7.71)	14 (5.11)	0.138	52 (6.05)	18 (7.38)	0.452	142 (7)	32 (6.18)	0.507
Renal disease, *n* (%)	159 (9.84)	68 (10.73)	0.530	192 (10.41)	88 (12.54)	0.125	351 (10.14)	156 (11.68)	0.121
AIDS, *n* (%)	6 (9.38)	0 (0)	NA	1 (5.88)	0 (0)	NA	7 (8.64)	0 (0)	NA

CCI: Charlson comorbidity index. AMI: acute myocardial infarction. CHF: congestive heart failure. PVD: peripheral vascular disease. CVD: cerebrovascular disease. COPD: chronic obstructive pulmonary disease. PUD: peptic ulcer disease. Liver disease: mild and moderate/severe liver disease. HP/PAPL: hemiplegia/paraplegia. The *p*-value for the difference between patients with T2DM and without T2DM was calculated with the bivariate unconditional logistic regression model.

**Table 4:** Multivariate analysis of variables associated with in-hospital mortality among men and women with type 2 diabetes mellitus hospitalized for pulmonary embolism in Spain (2016–2018).

	Men	Women	Both
	OR (95%CI)	OR (95%CI)	OR (95%CI)
40–59 years	1	1	1
60–74 years	1.55 (0.93–2.59)	0.73 (0.41–1.29)	1.19 (0.81–1.75)
>75 years	2.48 (1.5–4.1)	1.29 (0.76–2.21)	1.97 (1.37–2.86)
CCI = 0	1	1	1
CCI 1–2	2.37 (1.65–3.42)	2.54 (1.89–3.42)	2.48 (1.97–3.12)
CCI > 2	2.89 (1.99–4.19)	3.29 (2.38–4.55)	3.11 (2.44–3.97)
Massive PE	4.49 (1.62–12.45)	4.52 (1.72–11.89)	4.50 (2.3–9.17)
Atrial fibrillation	1.56 (1.13–2.16)	1.73 (1.29–2.3)	1.63 (1.32–2.03)
Obesity	0.55 (0.36–0.85)	0.68 (0.51–0.92)	0.64 (0.5–0.81)
Women	NA	NA	1.08 (0.80–1.41)

CCI: Charlson comorbidity index. PE: pulmonary embolism. The value of the Hosmer–Lemeshow goodness-of-fit statistic was 13.58, 11.47 and 11.17 (men, women, both) and the corresponding *p*-values were 0.093, 0.089 and 0.1921. This indicates that the models seem to fit quite well.

## Supplementary Materials

The following are available online at https://www.mdpi.com/1660-4601/17/22/8347/s1, Table S1: ICD-10 codes for the clinical diagnosis and procedures used in this investigation. Table S2: In-hospital mortality according to specific comorbid conditions and procedures variables among men and women with and without type 2 diabetes (T2DM) hospitalized with pulmonary embolism in Spain (2016–2018). Table S3. Prevalence of thrombolytic therapy and inferior vena cava (IVC) filter placement among patients hospitalized with or without massive pulmonary embolism (PE) according to sex and type 2 diabetes (T2DM) status in Spain (2016–2018). Table S4. Distribution and in-hospital mortality (IHM) of atrial fibrillation according to its presence at admission among patients hospitalized with PE and with and without T2DM.
