# Supplementary material for: Type 2 Diabetes Is a Risk Factor for Suffering and for in-Hospital Mortality with Pulmonary Embolism. A Population-Based Study in Spain (2016–2018)
Source: Int J Environ Res Public Health. 2020 Nov 11;17(22):8347. doi: 10.3390/ijerph17228347 (PMC7698274; doi:10.3390/ijerph17228347)
Supplement: Supplementary file 1 [file ijerph-17-08347-s001.pdf]

Supplementary Table 1. ICD-10 codes for the clinical diagnosis and procedures used in this investigation.

Variable	ICD-10
Valvular heart disease	I05.X, I06.X, I07.X, I08.X, I34.X,I35.X, I36.X, I37.X
Hypertension	I10, I16.6
Obesity	E66.X
Coagulopathy	D68.XX
Non-septic shock	R57.0, R57.1, R57.8, R57.9,
Invasive mechanical ventilation	5A1945Z, 5A1955Z, 5A1935Z
Non-invasive mechanical ventilation	5A09357, 5A09457, 5A09557
Thrombolytic therapy	3E03317, 3E04317, 3E05317, 3E06317, 3E08317
Inferior vena cava filter placement	02HV0DZ, 02HV3DZ, 02HV4DZ, 02LV0CZ, 02LV0DZ, 02LV0ZZ, 02LV3CZ, 02LV3DZ, 02LV3ZZ, 02LV4CZ,,02LV4DZ,,02LV4ZZ,,02VV0CZ , 02VV0DZ, 02VV0ZZ, 02VV3CZ, 02VV3DZ , 02VV3ZZ, 02VV4CZ, 02VV4DZ , 02VV4ZZ , 6H00DZ, 06H03DZ , 06H04DZ , 06L00CZ, 06L00DZ , 06L00ZZ , 06L03CZ, 06L03DZ , 06L03ZZ , 06L04CZ, 06L04DZ , 06L04ZZ , 06V00CZ, 06V00DZ ,06V00ZZ 06V03CZ, 06V03DZ, 06V03ZZ, 06V04CZ, 06V04DZ, 06V04ZZ
Vasopressors	3E030XZ, 3E033XZ, 3E040XZ, 3E043XZ, 3E050XZ, 3E053XZ, 3E060XZ, 3E063XZ

Supplementary Table 2. In-hospital mortality according to specific comorbid conditions and procedures variables among men and women with and without type 2 diabetes (T2DM) hospitalized with pulmonary embolism in Spain (2016-2018).

	**Men**			**Women**			**Both**		
	**No T2DM**	**T2DM**	**p-value**	**No T2DM**	**T2DM**	**p-value**	**No T2DM**	**T2DM**	**p-value**
Massive PE, n (%)	274(42.15)	69(36.7)	0.181	249(37)	63(38.65)	0.696	523(39.53)	132(37.61)	0.511
Valvular heart disease, n(%)	77(7.94)	22(11.58)	0.103	100(6.49)	26(8.87)	0.142	177(7.05)	48(9.94)	0.028
Atrial fibrillation, n (%)	226(14.13)	64(16.37)	0.260	246(12.19)	80(16.63)	0.010	472(13.05)	144(16.51)	0.008
Hypertension, n(%)	425(6.54)	164(8.4)	0.005	558(6.21)	205(7.97)	0.002	983(6.35)	369(8.15)	<0.001
Obesity, (n%)	64(4.04)	27(4.78)	0.457	116(4.17)	66(6.77)	0.001	180(4.12)	93(6.04)	0.002
Coagulopathy, n(%)	15(4.3)	1(2.38)	0.559	19(6.4)	6(11.54)	0.191	34(5.26)	7(7.45)	0.390
Undergone surgery, n(%)	70(18.62)	18(23.68)	0.310	55(15.9)	14(28)	0.038	125(17.31)	32(25.4)	0.032
Thrombolytic therapy, n(%)	87(8.07)	15(8.02)	0.982	92(8.22)	26(11.5)	0.113	179(8.15)	41(9.93)	0.233
IVC filter placement, n(%)	13(7.03)	5(17.24)	0.075	15(8.24)	1(3.7)	0.421	28(7.63)	6(10.71)	0.431

CTPA: computed tomographic pulmonary angiography. IVC: inferior vena cava. LOHS: Length on hospital stay. IHM: In-hospital mortality. The P value for the difference between patients with T2DM and No T2DM was calculated with the bivariate unconditional logistic regression model

Supplementary Table 3. Prevalence of thrombolytic therapy and Inferior Vena Cava (IVC) filter placement among patients hospitalized with or without massive pulmonary embolism (PE) according to sex and type 2 diabetes (T2DM) status in Spain (2016-2018).

			Thrombolytic therapy			IVC filter placement		
			NO n (%)	YES n (%)	p-value	NO n (%)	YES n (%)	p-value
Male	No massive PE	Non T2DM	16861(94.67)	950(5.33)	0.181	17647(99.08)	164(0.92)	0.323
		T2DM	3193(95.23)	160(4.77)		3328(99.25)	25(0.75)	
	Massive PE	Non T2DM	522(80.31)	128(19.69)	0.097	629(96.77)	21(3.23)	0.434
		T2DM	161(85.64)	27(14.36)		184(97.87)	4(2.13)	
Female	No massive PE	Non T2DM	19268(95.12)	989(4.88)	0.944	20095(99.2)	162(0.8)	0.206
		T2DM	3894(95.09)	201(4.91)		4070(99.39)	25(0.61)	
	Massive PE	Non T2DM	543(80.68)	130(19.32)	0.241	653(97.03)	20(2.97)	0.212
		T2DM	138(84.66)	25(15.34)		161(98.77)	2(1.23)	
Both	No massive PE	Non T2DM	36129(94.91)	1939(5.09)	0.374	37742(99.14)	326(0.86)	0.107
		T2DM	7087(95.15)	361(4.85)		7398(99.33)	50(0.67)	
	Massive PE	Non T2DM	1065(80.5)	258(19.5)	0.045	1282(96.9)	41(3.1)	0.161
		T2DM	299(85.19)	52(14.81)		345(98.29)	6(1.71)	

PE Pulmonary embolism. P values obtained using bivariate unconditional logistic regression model.

Supplementary table 4. Distribution and in hospital mortality (IHM) of atrial fibrillation according to its presence at admission among patients hospitalized with PE and with and without T2DM

ATRIAL FIBRILLATION	Distribution			IHM			TOTAL POPULATION		
	T2DM	NO T2DM	P value for distribution	T2DM	NO T2DM	P value for IHM	Distribution	IHM	P value for IHM
Not present on admission. N (%)	111(12.73)	470(12.99)	0.837	19(17.12)	68(14.47)	0.482	581(12.94)	87(14.97)	0.346
Present on admission N (%)	761(87.27)	3148(87.01)		125(16.43)	404(12.83)	0.009	3909(87.06)	529(13.53)	

P values obtained using bivariate unconditional logistic regression model.
